# Low-cost prediction of molecular and transition state partition functions *via* machine learning†

**DOI:** 10.1039/d2sc01334g

**Published:** 2022-06-14

**Authors:** Evan Komp, Stéphanie Valleau

**Affiliations:** Chemical Engineering, University of Washington 3781 Okanogan Ln Seattle WA 98195 USA evankomp@uw.edu valleau@uw.edu

## Abstract

We have generated an open-source dataset of over 30 000 organic chemistry gas phase partition functions. With this data, a machine learning deep neural network estimator was trained to predict partition functions of unknown organic chemistry gas phase transition states. This estimator only relies on reactant and product geometries and partition functions. A second machine learning deep neural network was trained to predict partition functions of chemical species from their geometry. Our models accurately predict the logarithm of test set partition functions with a maximum mean absolute error of 2.7%. Thus, this approach provides a means to reduce the cost of computing reaction rate constants *ab initio*. The models were also used to compute transition state theory reaction rate constant prefactors and the results were in quantitative agreement with the corresponding *ab initio* calculations with an accuracy of 98.3% on the log scale.

## Abstract 2

Deep neural networks accurately predict transition state partition functions at the low cost of reactant and product input features for organic chemistry gas phase reactions.

## Graphical abstract

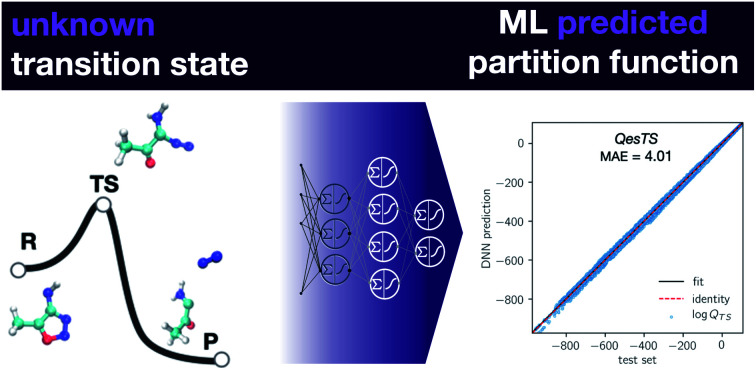

## Introduction

The reaction rate constant or speed of a chemical reaction defines its success. Evaluating reaction rate constants in organic chemistry is critical for drug design, catalyst design, and so forth. Unfortunately, reactions are usually part of large chemical networks. The challenge thus becomes evaluating the kinetics of these networks, *i.e.* all single reaction rate constants must be computed. The full *ab initio* calculation of a reaction rate constant can take several months of computational and human time.^1^ Indeed, most kinetic theories require the exploration of one or more potential energy surfaces and rely on the evaluation of a reactant and transition state partition function.^2–4^ These can only be computed when the structure of the corresponding species is known. In many cases, *e.g.* transition state theory (TST)^5^ the kinetics is represented by a single minimum energy path (MEP) which passes through a transition state. Finding the transition state structure is the most demanding part of the computation. Indeed, most MEP search methods^6^ require the iterative evaluation of gradients and energies of intermediate geometries until an MEP is found. Lastly even converged MEPs are not always guaranteed to include a true transition state and one must use *e.g.* eigenvector following to ensure a first order saddle point is found. In this context, using machine learning (ML) to predict any information regarding the transition state without knowledge of its structure is of strong interest.

Once input features have been computed, a trained machine learning estimator may predict chemical properties within seconds.^7–15^ Hence, in the last few years several efforts have been made to leverage ML for kinetics.^21^ Models have been trained to predict reaction energies,^22^ bond dissociation energies^21^ and recently activation energies.^23,24^ Machine learning has also been used to predict reaction products,^25–28^ yields^29,30^ or ideal synthesis conditions.^31^ Recently ML estimators were trained to predict quantum reaction rate constants for one dimensional minimum energy paths.^20,32^ All of the above is encouraging and only limited in application by the size of the training datasets and lack of large kinetic datasets.^19^ ML has also been used to help accelerate the search for MEPs^16–18^ and recently larger datasets of transition states^33,34^ and quantum rate constants^20^ have become available.

**Fig. 1:**
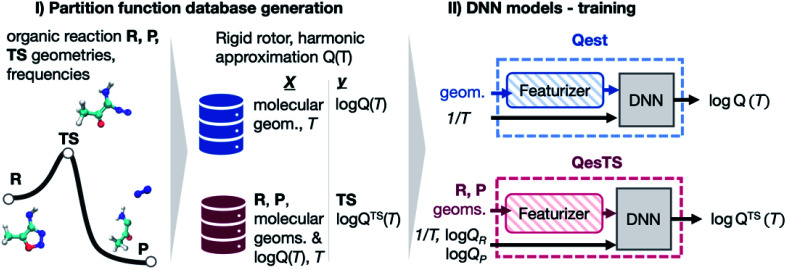
(Panel I) Partition functions were computed for a set of DFT optimized reactant, product, and transition state geometries taken from a dataset of 11 961 unimolecular reactions.^36^ (Panel II) With this data, a deep neural network (DNN), *Q*_est_, was trained to predict a structure's partition function from its geometry and the inverse temperature, 1/*T*. A second DNN, *QesTS*, was trained to predict the partition function of an unknown transition state from the reactant and product structures, partition functions, along with 1/*T*.

In this work we investigated the use of ML to predict transition state partition functions without knowledge of its geometry as well as predicting partition functions when geometries are known (Fig. 1). Together with an activation energy predictor,^23,24^ an accurate ML predictor of partition functions could circumvent the need to find a transition state structure when using TST to compute reaction rate constants. This would enable the rapid evaluation of kinetics for large networks of reactions. In addition, quantum reaction rate constant theories such as the flux–flux autocorrelation function still need the reactant partition function.^35^ Hence this predictor would help accelerate the calculation of quantum reaction rate constants. Lastly, it could be used to accelerate the evaluation of all other thermodynamic quantities which rely on knowledge of the partition function: free energy, entropy, pressure, heat capacity, *etc.* We generated a dataset of over 30 k partition functions for gas phase organic chemistry species extracted from an existing dataset^36^ of reactant, product, and transition state structures for unimolecular reactions. With this data, two deep neural network (DNN) partition function estimators were optimized. The first DNN, “*Qest*”, predicts the natural logarithm of the molecular partition function from featurized molecular geometry and inverse temperature, 1/*T*. The second DNN, “*QesTS*”, predicts the natural logarithm of the partition function of an unknown transition state, log *Q*_TS_(*T*) from the difference between product and reactant featurized geometries, the reactant and product partition functions on the log scale, and 1/*T*. These models were then used to predict partition functions to be used in the computation of transition state theory (TST) reaction rate constants. Our reaction rate constant predictions were also compared to *ab initio* TST reaction rate constants. The various steps of our workflow, our results, and conclusions will be discussed in the following subsections.

## Results and discussion

### Generation of a partition function dataset

Canonical partition functions were computed for 35 883 reactant, product, and transition state geometries taken from a dataset^33^ which contained 11 961 gas phase, organic chemistry reactions involving molecules of at most seven C, O and or N atoms. The partition functions were computed under the assumption that electronic, translational, rotational, and vibrational degrees of freedom were separable (eqn (1)), and by using the rigid-rotor and harmonic-oscillator approximations:

1 *Q*(*T*) ≈ *Q*_el_(*T*)*Q*_trans_(*T*)*Q*_rot_(*T*)*Q*_vib_(*T*).

See Table S1 in the ESI† for the equations of each partition function term in eqn (1). Although some products consisted of two or more distinct molecules, these had been considered as single structures when energies and hessians were computed in the original dataset.^36^ We used the single geometries and vibrational frequencies from ref. 33 to compute the partition functions. A more accurate representation would require the separation of the product geometry into the individual molecular geometries. When computing the vibrational partition functions, zero-point energies were included. For the rigid rotor rotational partition function, we computed the moments of inertia using the geometries and determined the symmetry number by identifying invariant symmetry operations using pymatgen,^37^ and by using our own software to determine which symmetry operations were proper. We accounted for the exception of linear molecules for which C_2_ rotations are returned as improper reflections by pymatgen.

**Fig. 2:**
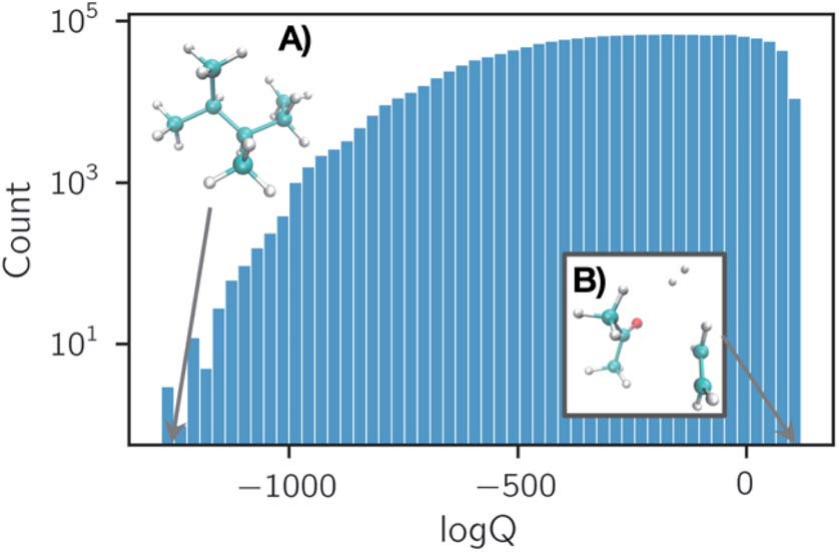
Histogram of all partition function values computed in our dataset for 35 883 organic chemistry species. Each entry is either a reactant, product, or transition state at one of 50 temperatures *T* ∈ (50, 2000) K, sampled uniformly with respect to 1/*T*. Structures that exhibit the smallest and largest partition functions in the dataset are depicted in subpanels (A) and (B). The shape of this histogram is largely due to the dominating contribution of the vibrational partition function in eqn (1).

Partition functions were computed over a set of 50 temperatures randomly sampled for each reaction from a uniform distribution of 1/*T* within the range of *T* = (50, 2000) K such that each reaction was considered at a unique set of temperatures. A histogram of the natural logarithm of the partition functions for all structures at all temperatures is shown in Fig. 2. The histogram shows a smaller count of low values of the natural logarithm of the partition function. This is due to the dominating contribution of the vibrational partition function to eqn (1). Our data has been made open source and is available to download from ref. 38.

The entire dataset was split into a hold out test set (10%) and a development set (90%). The hold out set was used to test final ML predictors. The development set was further split into 5 folds to use for cross validation during model optimization. Both the hold-out and the fold splitting were conducted using the Murcko scaffold of the reactant molecule.^39^ Here the scaffold is a representation of the structural backbone of the molecule. In splitting by scaffold we ensured that the same and similar molecules in the dataset were grouped together, producing a better estimate of the models accuracy on unseen data when conducting cross validation or testing. For *Qest*, the input consisted of a molecule's featurized structure (reactant, product, or transition state) and the inverse temperature, while the target was the corresponding natural logarithm of the partition function at that temperature. On the other hand, for *QesTS* the inputs were the difference between product and reactant featurized structures, product and reactant partition functions, and the inverse temperature while the target was the natural logarithm of the transition state partition function.

### Optimization and training of machine learning models

We first determined the optimal format of the input data using the cross validated development set. This involved a screening of featurization methods and data standardization. Only 3D structure featurizers were investigated because 2D featurizers require bond connectivity which is arbitrary for unstable structures such as transition states. Furthermore, 2D featurizers cannot resolve for orientation differences in 3D geometries such as those coming from different local minima for reactants. This means that 2D featurizers cannot be used for the *Qest* model. Congruently, we note that the partition functions and rate constants we computed in this work only consider the given reactant or product local minima for each reaction from the original dataset. To compute a more accurate reaction rate constant, multiple local minima would need to be considered. We chose to use the difference in product and reactant input feature vectors as it describes the change in 3D structure for the reaction. Further, product and reactant feature differences have shown to be successful when training other ML models to predict reaction quantities.^19,23,24,40^ Concatenating feature vectors may also be effective, but would require a larger DNN.

We found that the EncodedBonds^41^ featurizer with min–max scaling and target normalization were optimal for the *Qest* Model. For the *QesTS* model we employed the same input data feature representation as *Qest*, *i.e.* Encoded Bonds. We also carried out the screening of data standardization and found it was better not to use standardization. We believe that this is because the distribution of values for the difference in product and reactant feature vectors is peaked around zero; also, the distribution of partition function values for transition states is narrower than the overall partition function distribution for all species. For more information see ESI, section 2.†

With these optimal input features, we carried out a search over DNN hyperparameters to identify the best activation function, regularization, bias and weight initialization, learning rate, and neuron configuration for *Qest* and *QesTS*. For more information see ESI, section 3.†

### Partition function prediction

The optimal featurizer, standardization, and hyperparameters (Table S4†) were used to train the final DNN models on the development dataset and test its performance on the test set. To limit extrapolation in the final model, some outliers were dropped from the total dataset, see ESI, section 5.†

**Fig. 3:**
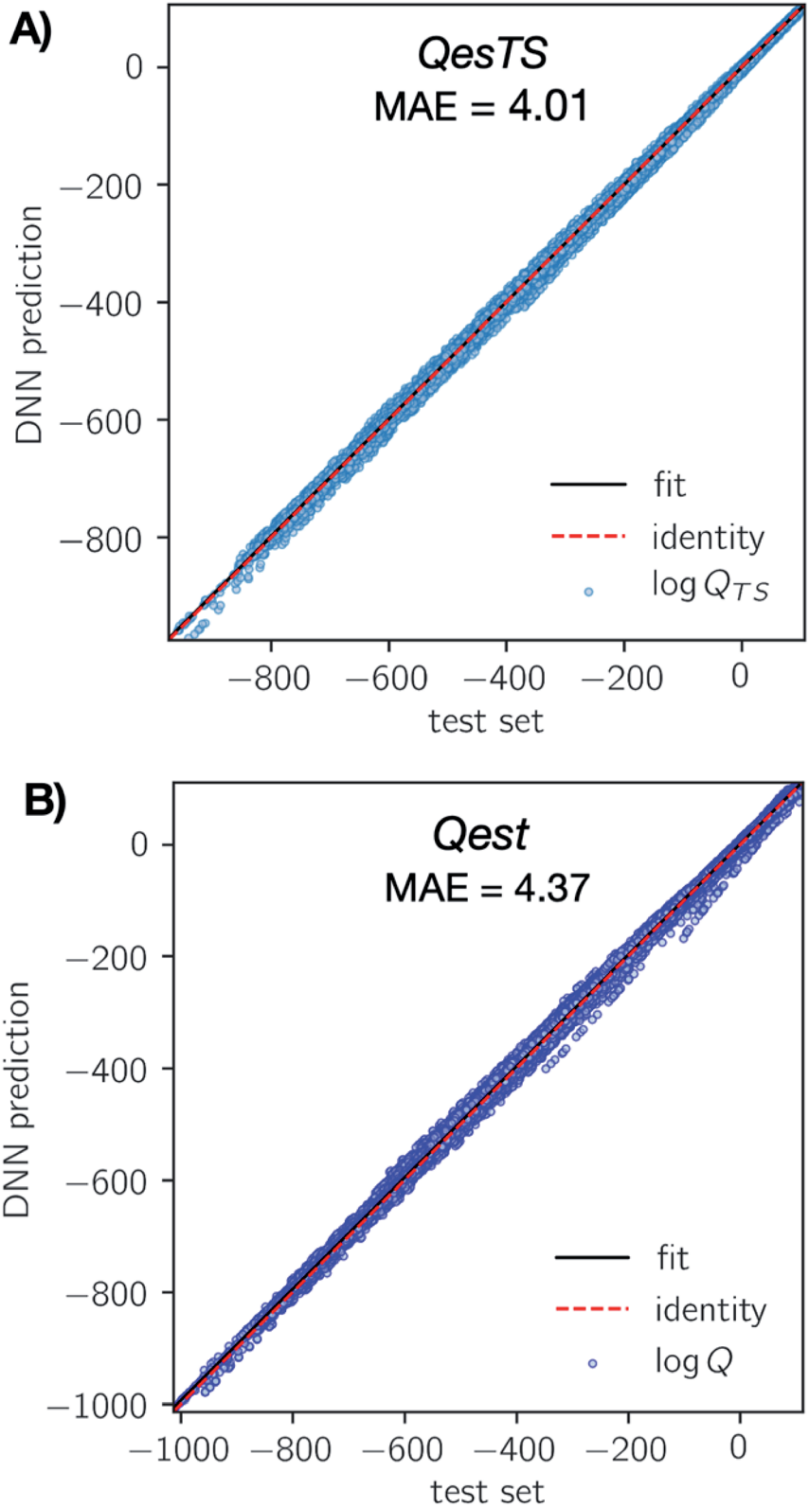
(Panel A) Parity plot of the final *QesTS* DNN predicted values of transition state log *Q*_TS_ (*y*-axis) with respect to exact unseen test set values (*x*-axis). A perfect model is represented by the identity line (red dashes). Predicted examples (blue dots) closely match the true value and the overall MAE on the log *Q* is 4.01 (2.0%). (Panel B) Same as panel A but for the *Qest* prediction of log *Q*. Here the overall MAE on the test set log *Q* is 4.37 (2.1%). Percent error is compared to the test set standard deviation. We see a strong agreement of our DNN predictions with the test set values.

In Fig. 3 we show parity plots of the final *QestTS* (panel A) and *Qest* (panel B) model predictions with respect to the test set. In both cases we see a low test set mean absolute error (MAE) of 4.01 (2.0%) and 4.37 (2.1%) on the logarithm of the chemical species or transition state partition function for *QesTS* and *Qest* respectively. Percentages are MAE compared to the test set standard deviation. Also, in both cases the spread of the distribution of predictions around the identity is quite narrow. The distribution of errors is shown in ESI, section 4.† This indicates that both DNN models accurately predict the partition functions.

To verify that the *Qest* model was learning from the molecular structures in the dataset and not simply from the temperature, we created a “null” linear model log *Q*(*T*) = *m* × 1/*T* + *b* and fitted it to the development set. The use of this null model for both reactant and transition state partition functions corresponds to the commonly used assumption that the ratio of partition functions is equal to 1.0. The null model performed poorly on the test set with an MAE of 31.8%. Our *Qest* model error was much lower: 1.9%, confirming that the *Qest* model learned from the molecular structure.

The prediction MAEs on the test set for *Qest*, *QesTS*, and the null model are listed in Table 1.

**Table 1:** Mean absolute error of the null, *Qest* and *QesTS* models in predicting the test set molecular (reactant, product and transition state) or transition state partition functions on the log scale. Percent error is given compared to the test set standard deviation

	Null MAE	*Qest* MAE	*QesTS* MAE
Log *Q*	72.5 (34.5%)	4.37 (2.1%)	N/A
Log *Q*_TS_	72.1 (35.1%)	4.25 (2.1%)	4.01 (2.0%)

Given that both models predicted with a low error on the test set, we combined these to predict transition state partition functions with machine learned reactant and product partition functions. We will discuss this “*Double*” model in the next section. The trained models are available on GitHub.^42^

### Reaction rate constants computed with machine learned partition functions

With our *Qest* and *QesTS* partition function estimators, and the existing energies,^36^ we computed transition state theory reaction rate constants for 1086 test set reactions. We recall the expression for the transition state theory,^43,44^ TST, reaction rate constant, 
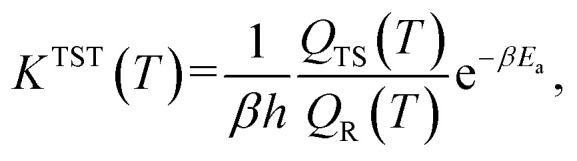
 where *E*_a_ is the activation energy as the difference in ground state energies between transition state and reactant, *β* = 1/*k*_B_*T* while *Q*_TS_ and *Q*_R_ are the transition state and reactant partition functions including zero point energy contribution at a given temperature *T*.

To understand how machine learned partition functions influence the accuracy of the reaction rate constant, three approaches were considered. In the first both reactant and transition state partition functions were predicted from their geometries by using *Qest*. In the second approach, the reactant and product geometries and computed *ab initio* partition functions were used to predict the transition state partition function with *QesTS*. This approach avoids the time demanding search for a transition state geometry. In the last approach, *Qest* was used to predict reactant and product partition functions which were used, together with their geometries, as input to *QesTS* to predict a transition state partition function. This method, hereafter referred to as the *Double* predictor, only requires knowledge of the reactant and product geometries and does not need computed partition functions or transition state geometries.

The MAE of the three methods for predicting test set transition state partition functions and TST reaction rate constants is listed in Table 2. For the prediction of transition state partition functions (Table 2, column 1) all models have a similar MAE. The *Double* approach is the least accurate with an MAE of 2.7% while the *QesTS* approach has the lowest MAE of 2%.

**Table 2:** Performance of transition state partition function prediction, and TST reaction rate constant calculation using predicted partition functions for our three ML based methods (*Qest*, *QesTS* and *Double*). Here *E*_a_ is the reaction activation energy. Percent error is given compared to the test set standard deviation

	Log *Q*_TS_ (*T*) MAE	Log *k*^TST^(*T*) MAE	Required inputs for *k*(*T*)
*Qest*	4.25 (2.1%)	4.50 (1.7%)	Reactant, transition state structures, temperature, *E*_a_
*QesTS*	4.01 (2.0%)	4.01 (1.5%)	Reactant, product structures and partition functions, temperature, *E*_a_
*Double*	5.58 (2.7%)	4.50 (1.7%)	Reactant, product structures, temperature, *E*_a_

The fact that *QesTS* is the best predictor is not surprising given that it was trained specifically to predict transition state partition functions while *Qest* was not. Further, *QesTS* has knowledge of reactant and product partition functions from its input. In column 2 of Table 2 we see that when computing reaction rate constants by using the predicted partition functions, the *Double* and *Qest* approaches have the same MAE while the *QesTS* approach remains the most accurate with an MAE of 4.01. The increase in accuracy of the *Double* approach compared to the other methods is most likely due to a cancelation of errors from *Qest* predicted reactant and *QesTS* predicted transition state partition functions. Nonetheless, we can achieve the same average accuracy of reaction rate constant calculations when using only reactant and product structures and no information on the transition state geometry. The only cost remains providing the value of the activation energy. Here it is worth noting that machine learning has successfully been employed to predict activation energies.^23,24^

**Fig. 4:**
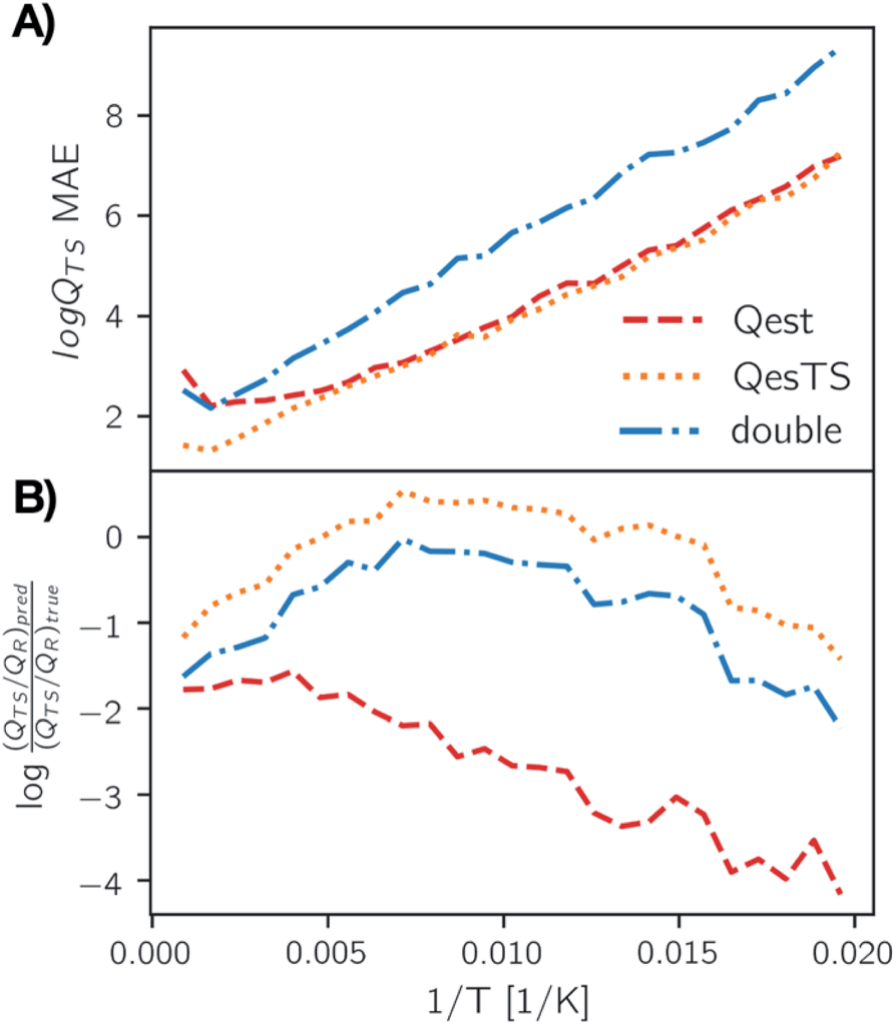
(Panel A) MAE averaged over temperature bins on the test set for predictions of the logarithm of the transition state partition function using *Qest*, *QesTS*, and *Double* models. While the error of the transition state logged partition function increases as temperature decreases, the ratio of transition state to reactant partition function, which is the prefactor for TST rate constants, has low error. This is shown in the plot of the logged ratio of true to predicted prefactors binned over temperature (panel B). Here 0.0 indicates a perfect match between predicted and true value.

**Fig. 5:**
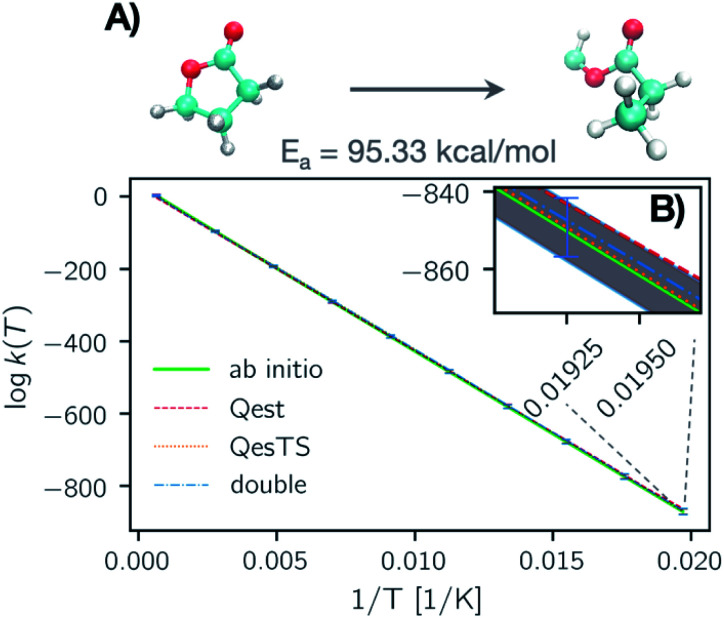
(Panel A) Natural logarithm of the reaction rate constant for a randomly selected test set reaction: the ring breaking of gamma-butyrolactone. Here we show the logarithm of the reaction rate constant computed with predicted *Qest* (red), *QesTS* (orange), and *Double* (blue) partition functions as well as the reaction rate constant computed using the *ab initio* values of the partition functions (green). The error bars correspond to the error on the entire test set averaged over temperature bins. (Panel B) A zoom in on the low temperature section of the data in plot A. Here the average error is plotted as dotted margins instead of error bars. One error bar (blue) is depicted. The error on *k*(*T*) for all prediction methods increases at low temperatures, however the average error is significantly smaller than the magnitude of the reaction rate constant.

In Fig. 4, panel A we plot the error of the predicted transition state partition function with respect to the test set partition functions as a function of temperature for *Qest*, *QesTS*, and *Double*. In panel B we show the logarithm of the ratio of predicted transition state theory prefactors, *Q*_TS_/*Q*_R_, with respect to computed prefactors. We see some error cancelation: the error in the ratio of partition functions (panel B) is smaller than that for the single values (panel A). Errors for all models tend to increase at lower temperatures even though low temperatures were sampled frequently when generating the data. This could come from the fact that the partition function is more sensitive to small changes in temperature at low temperatures, making it more difficult to learn. Regardless, these errors do not significantly impact the predicted value of the reaction rate constant, with average error orders of magnitude lower than the value of the logarithm of the reaction rate constant. This is also seen in Fig. 5, where we show the average error compared to the reaction rate constant for the ring breaking of gamma-butyrolactone.

**Fig. 6:**
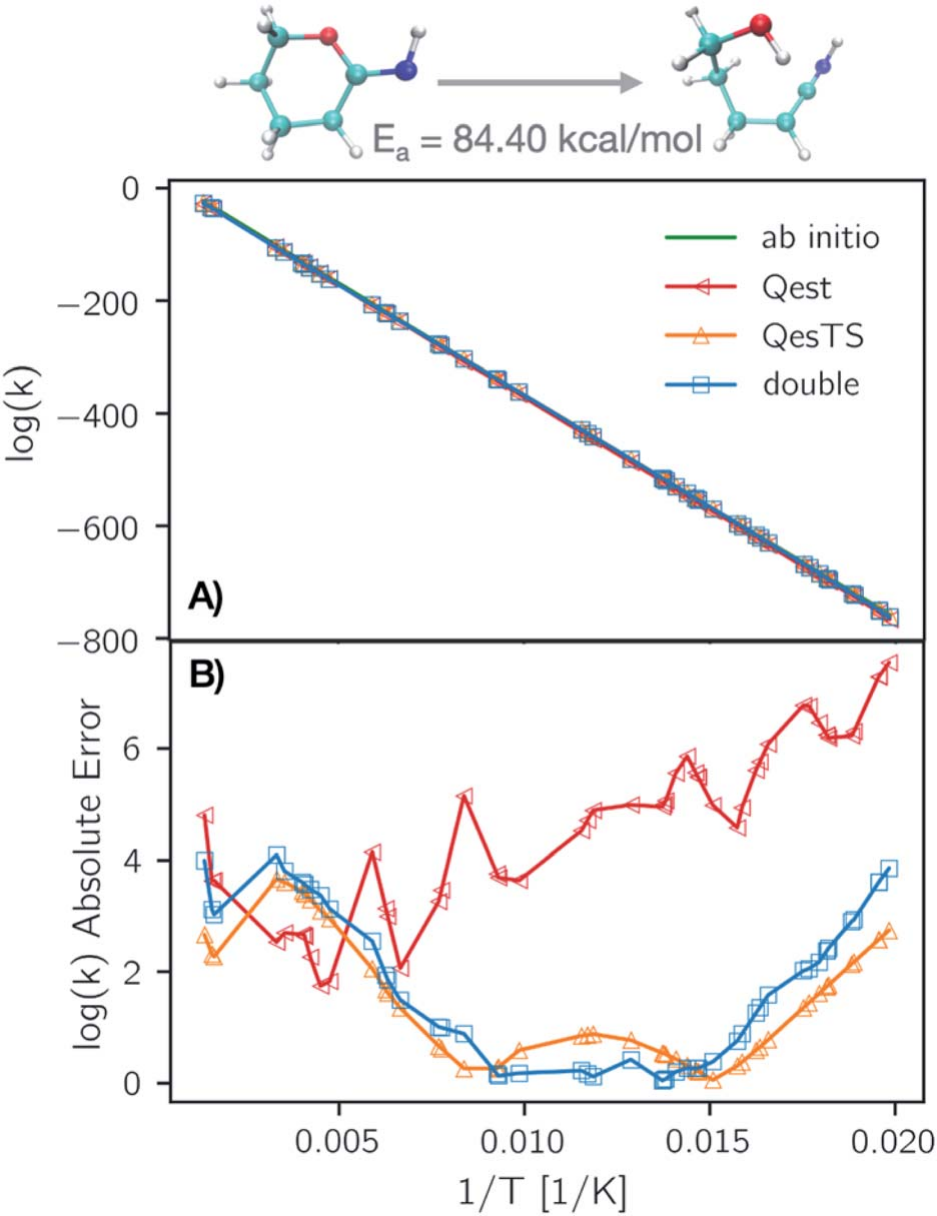
(Panel A) Natural logarithm of the TST reaction rate constant as a function of inverse temperature, 1/*T*, using *Qest*, *QesTS*, and *Double* predicted and computed partition functions for the ring opening of tetrahydro-2*H*-pyran-2-imine. This reaction was randomly selected from the test set. The predicted values are all close to the *ab initio* values. The absolute error of these models on the logarithm of the rate constant is shown in (panel B). *Qest* prediction has larger error at low temperatures for this reaction, however all methods predict with error an order of magnitude lower than the logarithm of the rate constant.

In Fig. 6 panel A, we show a plot of the transition state theory reaction rate constant for another reaction randomly selected from the test set, the ring opening of tetrahydro-2*H*-pyran-2-imine. Here the partition functions were computed using the original *ab initio* data (green line) and predicted with the ML models. In panel B, we show the absolute error as the normed difference between true and predicted values. We see that the error of the models is more than an order of magnitude smaller that the value of the logarithm of the reaction rate constant which confirms the accuracy of our trained models.

## Conclusion

In this work we have computed rigid rotor, rigid body, harmonic oscillator partition functions over a broad range of temperatures for a dataset of 11 961 organic chemistry unimolecular reactions with reactant, transition state, and product structures. With this dataset two DNN based models were trained to predict partition functions at given input temperatures. *Qest* predicts a molecular partition function from the molecule's featurized geometry, *QesTS* predicts transition state partition functions from reactant and product featurized geometries and their partition functions; lastly *Double* uses *QesTS* to predict *Q*_TS_ with *Qest* estimated reactant and product partition functions as well as reactant and product geometries. The *Double* approach requires no knowledge of the transition state structure; it only requires reactant and product structures. We showed that these estimators are accurate in their prediction and the MAE on the logarithm of the partition function is on the order of 2% (Table 1) for structures containing no more than 7 C, N, and O atoms at temperatures between 50 K and 2000 K. With these predicted partition functions, we computed transition state theory reaction rate constants and found an MAE of 1.6 ± 0.1% on log *k*(*T*) (Table 2). Predictions of unseen test set reactions such as the ring breaking of tetrahydro-2*H*-pyran-2-imine closely followed the exact test set values. For predictions outside of the temperatures explored here or structures involving more atoms, we recommend retraining. Indeed the model's accuracy can only be evaluated within the testing range.^45,46^ In the future, we plan to generate datasets for systems with more atoms, and to evaluate anharmonic partition functions to train new estimators. Transfer^47^ or delta^48^ learning might be used to accelerate these tasks.

The models we have created in tandem with activation energy predictors provide an approach to predict reaction rate constants without the need to search for minimum energy paths. Our models enable a more rapid estimation of reaction dynamics in the context of coupled reactions, reaction networks, and reactor design. We also would like to note that very recent work has used ML to predict transition state structures directly.^49,50^ Should these models become accurate on a broader scale they could provide an alternative path towards predicting TST rate constants.

In the future, we aim to move beyond unimolecular reactants and consider bimolecular reactions as well as reactions which occur in the presence of a solvent. We also plan to investigate other machine learning approaches, for instance, on employing end-to-end message passing models, given their recent success for the prediction of adjacent molecular and reaction quantities.^51,52^

## Data availability

The partition function dataset can be found on Zenodo.^38^ The trained ML estimators are available on GitHub.^42^

## Author contributions

E. K. and S. V. generated the software to compute the rotation symmetry number and partition functions. E. K. carried out the calculations for the partition function dataset and machine learning estimators. E. K. and S. V. generated and analyzed the results and wrote the manuscript and supporting information.

## Conflicts of interest

There are no conflicts to declare.

## Supplementary Material

SC-013-D2SC01334G-s001
