# Supplementary material for: Low-cost prediction of molecular and transition state partition functions via machine learning
Source: Chem Sci. 2022 Jun 14;13(26):7900–6. doi: 10.1039/d2sc01334g (PMC9258343; doi:10.1039/d2sc01334g)
Supplement: SC-013-D2SC01334G-s001 [file SC-013-D2SC01334G-s001.pdf]

Electronic Supplementary Material (ESI) for Chemical Science.
This journal is © The Royal Society of Chemistry 2022

Supplementary Information for "Low-cost prediction of molecular and transition state partition functions via machine learning"

Evan Komp*and Stéphanie Valleau*,†

(*) *Department of Chemical Engineering, University of Washington, Seattle, Washington 98195, United States*

† Corresponding authors: evankomp@uw.edu, valleau@uw.edu

Table of Contents

1 Dataset 1
2 Featurization 2
2.1 Search for optimal input features 2
2.2 Featurization parameters 3
2.2.1 DScribe Software 3
2.2.2 MolML Software 4
3 Optimization of hyperparameters 7
4 Outliers 7
5 References 8

1 Dataset

In **Figure S1** we show the activation energies of the reactions in the dataset used to generate the partition function dataset. In **Table S1** we show the partition function equations employed to compute $Q(T)$.

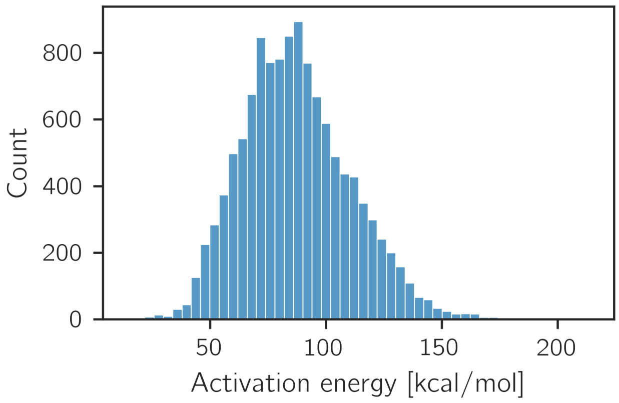

Figure S1: Histogram of ground state activation energies of all reaction used to generate the partition functions dataset. The average value of the activation energies $\langle E_a \rangle = 85.3$ kcal/mol is much higher than that of most organic chemistry reactions because these reactions involve breaking covalent bonds in unimolecular reactants.

Table S1: Equations employed to compute the translational, vibrational, and rotational partition functions with the rigid body, harmonic oscillator, and rigid rotor approximations. Here m is the molecular mass, V the volume, ω are the wavenumbers, $\theta_i = h^2/8\pi^2 I_i$ where I_i is the moment of inertia and σ is the symmetry number. The electronic partition function was approximated as equal to the electronic ground state degeneracy, $g_{el,0}$.

	Partition function	
**Translational**	$Q_{trans} = V\left(\frac{\sqrt{2\pi m k_B T}}{h}\right)^3$	
**Vibrational**	$Q_{vib} = \prod_{i=1}^{3N-6} \frac{e^{-\hbar\omega_i/2k_B T}}{1-e^{-\frac{\hbar\omega_i}{k_B T}}}$	
**Rotational**	$Q_{rot} = \frac{\sqrt{\pi}}{\sigma}\sqrt{\frac{(k_B T)^3}{\theta_x\theta_y\theta_z}}$	3D structure
	$Q_{rot} = \frac{k_B T}{\sigma\theta}$	linear structure

2 Featurization

2.1 Search for optimal input features

For the *Qest* model, we screened over a series of geometry based input features which included Autocorrelation (AC) [1], EncodedBonds (EB) [2], and Coulomb Matrix (CM)[3] as well as Smooth Overlap of Atomic Positions (SOAP) [4], and Many Body Tensor Representation (MBTR) [5]. To create these, the MolML [2] and DScribe [6] software packages were used. See section 2.2. Graph based featurization techniques were not considered and this is in plan for future work. For each featurization we tested feature and target standardization; we considered min-max scaling and gaussian normalization. The model hyperparameters used during this screening are shown in **Table S2**.

Overall, we searched over 45 possible combinations of input feature, feature standardization, and target standardization and found that EncodedBonds with feature min-max scaling and target normalization were optimal. The average performance of the different featurizers is shown in **Figure S2**. We repeated the screening of standardization for *QesTS* using EncodedBonds, which yielded no standardization as optimal.

Table S2: Hyperparameters used during data screening.

Hyperparameter	Value
layer sizes	[200, 200]
learning rate	0.001
batch size	100
epochs	20
hidden activation function	relu
l2 regularization	0.0

bias initialization constant	1.0
weight initialization stdev	0.2
loss	mean squared error

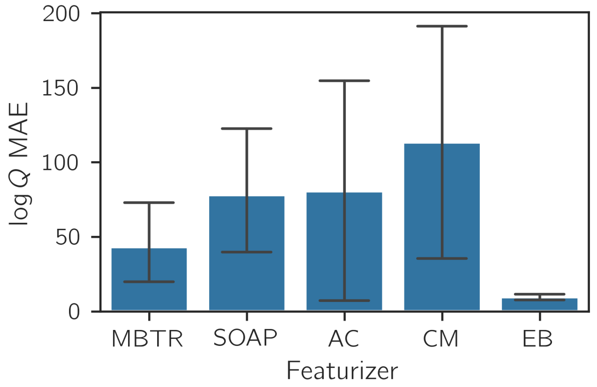

Figure S2: Average performance of each featurizer tested during screening. Bars represent the cross validated mean absolute error, while error bars are the standard deviation across the data standardization combinations tested for each featurizer. Models using Encoded Bonds performed best on average. Also, the best performing combination used Encoded Bonds with feature min-max scaling and target normalization.

2.2 Featurization parameters

The parameters used to produce the input features are described in subsection 2.2.1 and 2.2.2. Smooth Overlap of Atomic Positions (SOAP) and Many-body Tensor Representation (MBTR) input features were produced with the DScribe software, while Autocorrelation, Encoded Bonds, and Coulomb Matrix were produced with the MolML software.

2.2.1 DScribe Software

The DScribe software package[6] was used to generate SOAP and MBTR input features. The parameters used are listed below.

SOAP – Smooth Overlap of Atomic Positions

The parameters used to produce SOAP features are listed in python dictionary format:

```
{
   'species': ["H", "C", "O", "N"],
   'rcut': 6.0,
   'nmax': 8,
   'lmax': 6,
```

```
  'average': 'inner'
}
```

For a description of these parameters, we refer the reader to Ref [6].

MBTR – Many-body Tensor Representation

The parameters used to produce MBTR features are listed hereafter in python dictionary format:

```
{
    species=["H", "O", 'C', 'N'],
    k1={
        "geometry": {"function": "atomic\_number"},
        "grid": {"min": 0, "max": 8, "n": 100, "sigma": 0.1},
    },
    k2={
    "geometry": {"function": "inverse\_distance"},
    "grid": {"min": 0, "max": 1, "n": 100, "sigma": 0.1},
    "weighting": {"function": "exponential", "scale": 0.5, "cutoff": 1e-3},
    },
    k3={
    "geometry": {"function": "cosine"},
    "grid": {"min": -1, "max": 1, "n": 100, "sigma": 0.1},
    "weighting": {"function": "exponential", "scale": 0.5, "cutoff": 1e-3},
    },
    periodic=False,
    normalization='none',
    flatten=True
}
```

We refer the reader to Ref [6] for a description of these parameters.

2.2.2 MolML Software

MolML [2] was used to define the EncodedBonds, Coulomb Matrix and Autocorrelation features. MolML determines parameters such as the element types present and the maximum number of atoms during featurization. Due to limited RAM, the entire dataset could not be loaded at once, so it was scanned for a set of 3 structures which contained both the full array of chemical elements in the dataset, as well as the largest molecular system. The featurizers were fit to this minimal set, and then used to transform the entire dataset in chunks. The minimum subset of systems is given below in xyz format and angstrom units:

C	-2.301345240000000	0.245555560000000	-0.146458820000000
O	-1.801117640000000	-1.068638690000000	-0.132535600000000
C	-0.698207700000000	-1.254313550000000	-0.983955260000000
C	0.498785910000000	-0.503501480000000	-0.581849450000000
C	1.474872740000000	0.110834400000000	-0.254355130000000
C	2.665120440000000	0.852752900000000	0.143128950000000
H	-2.602697440000000	0.544988140000000	-1.159271040000000
H	-3.175412910000000	0.262006730000000	0.502576870000000
H	-1.561197990000000	0.962926400000000	0.225284290000000
H	-0.479999560000000	-2.323300650000000	-0.969310430000000
H	-0.961290100000000	-0.982075860000000	-2.016315420000000
H	3.153957870000000	0.378701800000000	0.995343110000000
H	3.380779340000000	0.898604960000000	-0.679097780000000
H	2.407752320000000	1.875459330000000	0.422868020000000

10

O	2.288293630000000	0.484583070000000	-0.121712670000000
C	1.127501880000000	0.193607830000000	-0.071145460000000
C	-0.184064230000000	0.924152480000000	0.259412420000000
C	-0.873748740000000	-0.432000910000000	-0.015729600000000
N	0.451962880000000	-0.971798030000000	-0.300035950000000
H	-0.242967130000000	1.286299400000000	1.283947400000000
H	-0.427416880000000	1.726023410000000	-0.434781960000000
H	-1.362208720000000	-0.885687600000000	0.848217390000000
H	-1.550559340000000	-0.441221400000000	-0.871782480000000
H	0.773206630000000	-1.883958250000000	-0.576389100000000

23

C	2.349176060000000	0.084494630000000	-0.905389770000000
C	1.627675400000000	0.053488250000000	0.436342020000000
C	0.236369270000000	-0.590909200000000	0.414770920000000
C	-0.342881300000000	-0.607355670000000	1.828561880000000
C	-0.712898880000000	0.033731390000000	-0.629030880000000
C	-0.976517400000000	1.521383910000000	-0.403857370000000
C	-2.030065150000000	-0.732800180000000	-0.733683520000000
H	2.385265370000000	-0.910486730000000	-1.357281740000000

H	3.377237770000000	0.431331870000000	-0.786465570000000
H	1.861357880000000	0.752318050000000	-1.618183410000000
H	2.239225610000000	-0.501941370000000	1.154397840000000
H	1.554048780000000	1.069878880000000	0.838340710000000
H	0.375865430000000	-1.636038860000000	0.106313200000000
H	0.341081900000000	-1.112555420000000	2.513879630000000
H	-0.495666310000000	0.405190570000000	2.210740010000000
H	-1.298151790000000	-1.131761590000000	1.876033720000000
H	-0.213934640000000	-0.065520660000000	-1.599015070000000
H	-1.615287910000000	1.919106830000000	-1.195418250000000
H	-1.487633380000000	1.698516490000000	0.545714450000000
H	-0.054749190000000	2.106284580000000	-0.401858700000000
H	-2.609548020000000	-0.389412500000000	-1.593433690000000
H	-1.858128490000000	-1.805335340000000	-0.854936010000000
H	-2.651841000000000	-0.591607940000000	0.153459620000000

3 Optimization of hyperparameters

After screening for input features and standardization (Section S2) we carried a hyperparameter optimization. The search space for hyperparameter optimization is shown in **Table S3**.
The Tree Parzen [7,8] sampler along with the median pruning algorithm was used to identify the optimal hyperparameters (see **Table S4**) with Optuna [9] using a 5 trial startup for both models. This corresponds to 1258 and 7685 completed trials for the *Qest* and *QesTS* model, respectively.
The number of epochs over which to train the model for the optimal hyperparameters was determined by using early stopping averaged over the 5 folds. From this we found that 39 and 27 epochs were optimal stopping points for the *Qest* and *QesTS* models respectively.

Table S3: Parameter space tested for hyperparameter optimization. Layer parameters such as bias initialization are applied to all hidden layers uniformly; the search space is restricted by not considering layers having different parameters. One to three total hidden layers are tested, and in each case all layers have the same number of neurons chosen from the range [2 – 2000]. MSE loss is used.

**Hyperparameter**	**Search space**	**Distribution**
layer sizes	1-3 layers, 2 - 2000 neurons	categorical choice
learning rate	(1E-5, 1E-1)	loguniform
batch size	(32, 8000)	uniform integer
hidden activation function	relu, tanh, softsign, sigmoid, softmax	categorical choice
l2 regularization	(0.0, 0.1)	uniform
bias initialization constant	(-1.0, 1.0)	uniform
weight initialization stdev	(1E-3, 1E+0)	loguniform

Table S4: Optimum hyperparameters for both the Qest and QesTS DNN models found from TPE hyperparameter optimizations using Encoded Bonds as input features.

**Hyperparameter**	**Qest DNN**	**QesTS DNN**
layer sizes	[816, 816]	[749, 749]
learning rate	1.58E-4	3.13E-4
batch size	34	2609
hidden activation	relu	relu
l2 regularization	8.68E-6	4.20E-5
bias initial value	-0.664	-0.207
weight initialization stdev	0.407	0.059

4 Model Performance

The distributions of error on the test sets for the Qest and QesTS models are shown below.

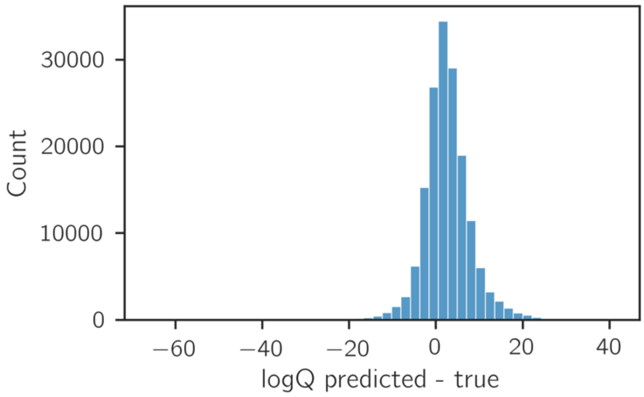

Figure S3: Distribution of errors on the natural logarithm of the partition function for the Qest model. Most errors cluster near 0, however there is a small bias towards overestimating the partition function.

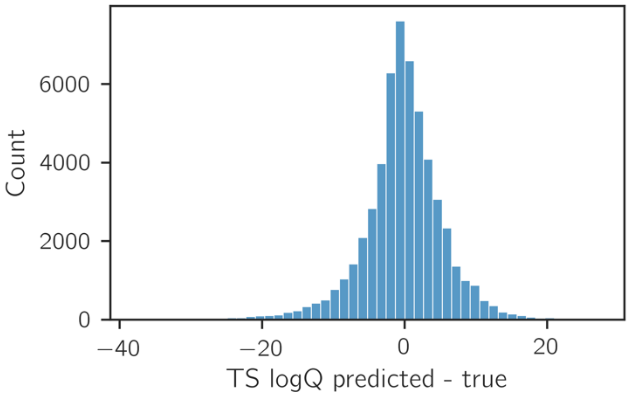

Figure S4: Distribution of errors on the natural logarithm of the transition state partition function for the QesTS model. Most errors cluster near 0. The model is unbiased, and the average error is -0.09.

5 Outliers

To limit extrapolation in the final model, outliers were dropped from the overall dataset as following. We identified reactions containing any example with 12 or more feature values ± 6 standard deviations away from that feature's mean. This resulted in removing 1,108 reactions.

6 References

1. J. P. Janet and H. J. Kulik, J. Phys. Chem. A **121**, 8939 (2017).
2. C. R. Collins, G. J. Gordon, O. A. Von Lilienfeld, and D. J. Yaron, J. Chem. Phys. **148**, 241718 (2018).
3. M. Rupp, A. Tkatchenko, K.-R. Müller, and O. A. von Lilienfeld, Phys. Rev. Lett. **108**, 058301 (2012).
4. A. P. Bartók, R. Kondor, and G. Csányi, Phys. Rev. B **87**, 184115 (2013).
5. H. Huo and M. Rupp, *Unified Representation of Molecules and Crystals for Machine Learning* (2017).
6. L. Himanen, M. O. J. Jäger, E. V. Morooka, F. Federici Canova, Y. S. Ranawat, D. Z. Gao, P. Rinke, and A. S. Foster, Comput. Phys. Commun. **247**, 106949 (2020).

7. J. Bergstra, D. Yamins, and D. D. Cox, **28**, (2013).
8. F. Hutter, L. Kotthoff, and J. Vanschoren, *Automated Machine Learning* (Springer, 2019).
9. T. Akiba, S. Sano, T. Yanase, T. Ohta, and M. Koyama, in *Proc. 25th ACM SIGKDD Int. Conf. Knowl. Discov. Data Min.* (ACM, New York, NY, USA, n.d.).
